# XMRV replicates preferentially in mucosal sites in vivo: Relevance to XMRV transmission?

**DOI:** 10.1186/1742-4690-8-S1-A219

**Published:** 2011-06-06

**Authors:** Francois Villinger, Jaydip Das Gupta, Nattawat Onlamoon, Ross Molinaro, Suganthi Suppiah, Prachi Sharma, Kenneth Rogers, Christina Gaughan, Eric Klein, Xiaoxing Qiu, Gerald Schochetman, John Hackett, Robert H Silverman

**Affiliations:** 1 Department of Pathology, Emory University of Medicine, Atlanta, GA, 30322, USA; 2 Division of Pathology Yerkes NPRC, Emory University, Atlanta, GA, 30322, USA; 3 Lerner Research Institute, Cleveland Clinic Foundation, Cleveland, OH, 44195, USA; 4 Mahidol University, Siriraj Hospital, Bangkok, Thailand; 5 Division of Pathology Yerkes NPRC, Emory University, Atlanta, GA, 30329, USA; 6 Abbott Diagnostics, Emerging Pathogens and Virus Discovery, Abbott Park, North Chicago, Illinois, 60064, USA

## 

Xenotropic Murine Leukemia Virus-related Retrovirus (XMRV) was identified from prostate cancer tissue using DNA based ViroChip technology as well as in a cohort of chronic fatigue syndrome patients. To delineate the infection dynamics and dissemination in vivo, we have thus far infected 7 healthy rhesus macaques and 2 pigtailed macaques. Results show that XMRV induces a chronic and clinically silent infection that is nevertheless persistent and susceptible to reactivation in vivo. While XMRV seems rapidly cleared from the blood circulation in healthy macaques, XMRV protein positive CD4+ T cells were detected in all lymphoid organs throughout infection. However, among all organs subjected to in situ detection, mucosal sites overall and sexual organs showed markedly higher frequencies of XMRVgag positive cells, including gastrointestinal mucosa, pulmonary environment and organs from the reproductive tract. Of interest, the lineage of cells that were XMRV positive markedly differed among the different sites, including CD4+ T cells in the GI mucosa, alveolar macrophages in the lung, epithelial cells in the prostate, seminal gland, vagina and cervix and interstitial cells in the testes. The latter were consistently observed during acute and chronic infection, suggesting the potential for sexual transmission of XMRV. In fact, a single atraumatic mucosal exposure with a high dose of XMRV virus into the urethra resulted in infection of 1 out of 4 macaques providing proof of concept that such transmission is possible. However, additional work is needed to fully investigate potential modes of XMRV infection.

